# Possible Factors Concerning the Crossing of Formosan Ferret-Badger Rabies over the Daan River in Miaoli County, Taiwan

**DOI:** 10.3390/ani15030319

**Published:** 2025-01-23

**Authors:** Cheng-Hung Lai, Mei-Chuan Wang, Chia-Ning Hsu, Chun-Yi Chang, Satoshi Inoue, Chang-Young Fei

**Affiliations:** 1 Department of Veterinary Medicine, College of Veterinary Medicine, National Chung Hsing University, Taichung 402202, Taiwan; 2 Department of Mathematics, College of Science, University of Taipei, Taipei 100234, Taiwan; margaret@go.utaipei.edu.tw; 3 Animal and Plant Health Inspection Agency (APHIA), Ministry of Agriculture, Taipei 100060, Taiwan; cnhsu@aphia.gov.tw; 4 Miaoli Animal Care and Health Office, Miaoli County 360017, Taiwan; changchunyi@ems.miaoli.gov.tw; 5 National Institute of Infectious Disease, Shinjuku-ku, Tokyo 162-8640, Japan; sinoue@niid.go.jp; 6 School of Veterinary Medicine, National Taiwan University, Taipei 106319, Taiwan; fei@ntu.edu.tw

**Keywords:** Daan river, Formosan ferret-badger, rabies, Taiwan

## Abstract

The first detection of Formosan ferret-badger (FFB) rabies on the main island of Taiwan occurred in July 2013. National surveillance reports indicated that prior to 2023, rabies outbreaks were limited to the southern region of the Daan River, which was considered a natural barrier preventing the spread northwards. However, in 2023, four new cases of rabid FFBs emerged in Miaoli County, located north of the Daan River, suggesting that the epizootic had crossed the river. Several factors may have contributed to the ability of reservoir FFBs to cross over the Daan River. Firstly, there was a decrease in rainfall in the upstream area of the Daan River in 2023. Secondly, the construction of the Shilin Weir by the Taiwan Power Company in the upstream area of the Daan River aimed to prioritize water diversion for hydropower generation while ensuring domestic water supply for Miaoli County and Taichung City. Lastly, there was an unusual increase in the number of roadkill FFBs during the rainy season of 2023. The decreased rainfall in FFB habitats in the mountains and the near-dry condition of the upstream Daan River caused by the Shilin Weir in 2023, allowing FFBs to easily cross the Daan River into Miaoli County. The evidence of unusually increased FFB activity in 2023 supports the speculation that FFBs crossed the Daan River, introducing FFB rabies into Miaoli County.

## Simple Summary

From the initial outbreak of Formosan ferret-badger (FFB) rabies in Taiwain in 2013 until December 2022, the epizootic had remained continuously limited on the south side of the Daan River, which was considered as a natural barrier preventing the reservoir FFBs from crossing over. However, in 2023, cases of FFB rabies appeared in Miaoli County, located north of the Daan River. Several factors may have contributed to this crossing, including (1) decreased rainfall in the Daan River catchment in recent years, reducing water sources in FFB habitats and forcing FFBs to seek forage outside their mountain habitats; (2) the construction of the Shilin Weir in the upstream of Daan River, possibly leading to drought during the dry seasons, allowing reservoir FFBs to cross from the south side into Miaoli County; and (3) thehe unusually high incidence of FFB roadkills s during the 2023 rainy season, indicating an overall increase in FFB activity. These pieces of evidence supported the speculation that FFBs crossed the Daan River, introducing FFB rabies into Miaoli County.

## 1. Introduction

Rabies is a kind of acute, progressive, and incurable viral encephalitis. The pathogen responsible for this disease belongs to the neurotropic RNA viruses of the genus Lyssavirus in the family Rhabdoviridae. The primary mammalian hosts of the virus are Carnivora and Chiroptera. The virus is mainly transmitted through animal bites, where the virus enters injured peripheral tissues and subsequently spreads to the central nervous system. Upon reaching the brain, it causes lethal encephalomyelitis [1]. Since 1999, the Animal and Plant Health Inspection Agency (APHIA) has established national animal rabies surveillance programs in Taiwan aimed at monitoring rabies in bats and wild carnivores.

**Figure 1:**
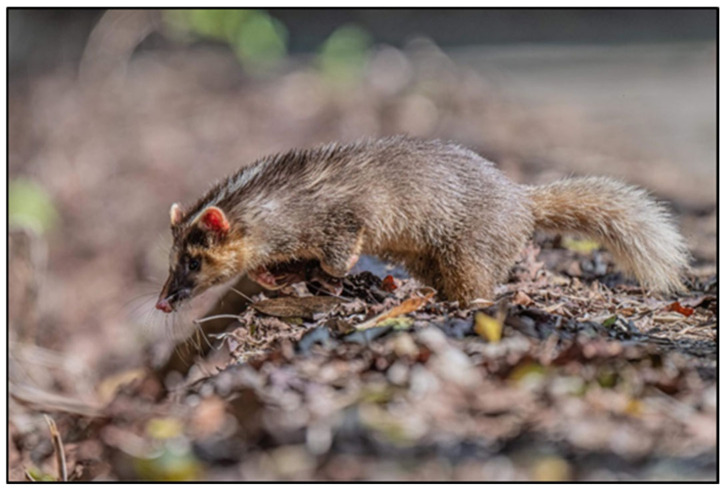
A released FFB under the trap–vaccinate–release program in Miaoli County (provided by Taichung Wildlife Conservation Society).

On 17 July 2013, Taiwan discovered cases of Formosan ferret-badger (FFB) rabies in wild FFBs and immediately notified the World Organization for Animal Health (WOAH) [2]. That was the first case of rabies in Taiwan since the World Health Organization declared Taiwan free from rabies in 1961 [3,4]. FFBs (Figure 1) predominantly feed on earthworms, insects, and berries [5], and generally weight less than 2 kg as an adult [6,7]. FFBs have limited vision, small teeth, and a weak bite force, making them unable to survive in areas with high populations of stray dogs [8]. FFBs are mainly found at the edges of forests, where they frequently forage in agricultural reclamation areas, industrial roads, or dry riverbeds in the dry season (from October to April) for the abundant earthworms, snails, insects, and berries. Additionally, FFBs do not scavenge human food or inhabit human dwellings [9]. As nocturnal animals, FFBs are distributed across different altitudes in Taiwan [10,11]. During the day, they rest in rock crevices and earthen caves. Conversely, during the rainy season (from May to September), FFBs have ample prey resources in the mountains and consequently venture out less frequently than during the dry season [12]. They have an average litter size of two and breed once a year [13]. Until 2023, there have been 12 cases of spillover infections from FFBs into non-reservoir hosts [14,15]. Currently, Taiwan is free of dog-mediated rabies, with FFBs being the only known rabies reservoir in the country, and no human deaths from FFB rabies have been reported.

FFB rabies was first detected on the main island of Taiwan in July 2013. National rabies surveillance reports have indicated that prior to 2023, FFB rabies outbreaks had been limited to the southern region of the Daan River, which acted as a natural barrier preventing northward spread [4,16,17,18]. Smith et al. [19] noted that rivers could impede the transmission of animal rabies epizootics, with wider rivers having a greater impact on delaying disease spread. However, in 2023, new cases of FFB rabies emerged in Miaoli County, located north of the Daan River [20].

The Daan River serves as a water source for Miaoli County and Taichung City, with the Shilin Weir playing a significant role in water diversion from the upstream area of the Daan River to the Zhuolan Hydropower Plant for hydroelectric power generation. Diverting water from the upstream regions often causes the riverbed to dry up. The rainfall in Taiwan has been declining for 40 years [21], which may have caused the upstream riverbed of the Daan River to dry up. In this study, we investigated the possible factors contributing to the spread of FFB rabies into Miaoli County through the analysis of inflow and outflow data from the Shilin Weir from 2013 to 2023, rainfall data from the Central Weather Administration, field investigations conducted by the authors in the upper reaches of the Daan River, and the record of roadkill FFBs in Miaoli County from year 2014 to 2023.

## 2. Materials and Methods

The national animal rabies surveillance program is a continuous plan aimed at monitoring and addressing cases of rabies. Under this plan, animals displaying suspected rabies signs are collected by municipal/prefectural competent authorities responsible for animal health, wildlife conservationist, researchers, and others involved. All collected samples are submitted to the National Veterinary Research Institute, Ministry of Agriculture, for rabies diagnosis [4]. The submitted samples are diagnosed using the direct fluorescent antibody (DFA) test, in accordance with the standard operating procedure [22]. In cases where the DFA test results are inconclusive, the sample is inoculated into the murine neuroblastoma cell line for rabies variant identification, as described by Tu et al. [17]. The Animal and Plant Health Inspection Agency (APHIA) conducted a comprehensive diagnosis, including RT-PCR, direct fluorescent antibody detection, immunohistochemistry (IHC), and antigen detection ELISA, as stated in the immediate notification (code: IN 13775) to the World Organization for Animal Health (WOAH) [2]. Sequencing and submission of products for comparison to the NCBI GeneBank are performed when conventional RT-PCR is required. Typically, the identified virus is the FFB rabies variant, which exhibits the highest genetic similarity to the samples. By the end of 2023, this program had inspected 5099 wild carnivores of which 2939 were FFBs and 922 were positive [23].

The wild carnivores in Taiwan primarily include FFBs, masked palm civets, crab-eating mongooses, small Indian civets, Siberian weasels, otters, Taiwan black bears, leopard cats, and martens [13]. Each municipal/prefectural competent authority for animal conservation assists the animal health section in accurately identifying the species of animal samples, which are then submitted to the National Veterinary Research Institute for rabies diagnosis.

The Animal and Plant Health Inspection Agency (APHIA) provides the location data of the four rabid FFBs collected in Miaoli County in 2023. The Taiwan Power Company supplied the monthly inflow and outflow data of the Shilin Weir from 2013 to 2023. The latitudes and longitudes of all objects in this study were obtained using Google Maps.

Rainfall analysis utilized observations from rain gauges at three conventional weather stations located in the upper reaches of the Daan River, operated by the Central Weather Administration in Taiwan. The Central Weather Administration provided monthly rainfall data from these stations between 2013 and 2023. The three stations were the Nanzhuang Weather Station, Nankuang Weather Station, and Madu-an Weather Station.

The data on monthly roadkill FFBs in Miaoli County from 2014 to 2023 were downloaded from the website of the Taiwan Roadkill Observation Network (TaiRON) [24]. TaiRON is a citizen science project launched by the Taiwan Biodiversity Research Institute, Ministry of Agriculture, in August 2011 to gain insights into animal deaths caused by transportation infrastructure and associated facilities (such as ditches, retaining walls, street lights, high-voltage wires, etc.), particularly casualties resulting from car accidents. The more common English terms are roadkill, road mortality, or wildlife vehicle collision. Rendall et al. [25] indicated that numbers of roadkill wildlife were associated with: (1) the abundance of wildlife and (2) traffic flow. According to this, if there were no factors concerning wildlife abundance and traffic flow, then these data could be used to evaluate the intensity of outdoor activities in FFBs. Additionally, the total length of roads was anticipated to vary from year to year, which may have disturbed the representation of the absolute value of roadkill FFBs. Consequently, we used the annual percentage of roadkill FFBs to evaluate the activity of FFBs.

## 3. Results

**Figure 2:**
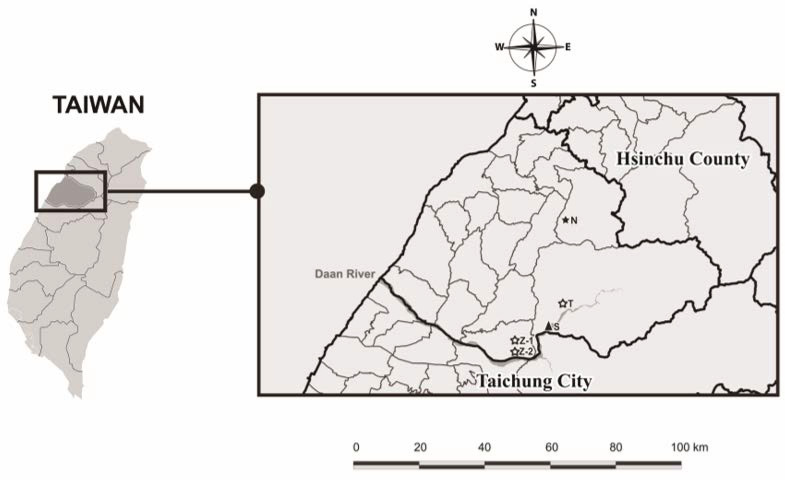
Location data of the sole triangle-coded Shilin Weir (S) and four star-coded rabid FFBs (N, T, Z-1, Z-2) in Miaoli County.

Figure 2 shows the locations of the Shilin Weir (S) in Miaoli County and the four rabid FFBs sampled in 2023. Three rabid FFBs (coded T, Z-1, and Z-2) were sampled in January 2023 upstream near the Daan River, whereas the fourth (coded N) was sampled in December 2023 upstream and further away from the Daan River. Additionally, Figure 2 shows that the Shilin Weir is upstream from the Daan River.

**Figure 3:**
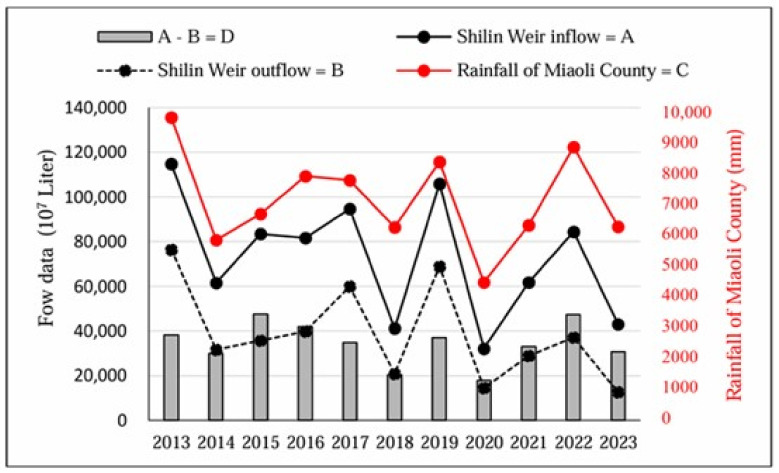
Annual plots of Shilin Weir inflow (A), Shilin Weir outflow (B), the total rainfall of weather stations at Miaoli County (C), and the value of A minus B (D) from 2013 to 2023. Data source: (1) Taiwan Power Company; (2) Central Weather Administration.

Figure 3 illustrates the results of the Shilin Weir prioritizing increased hydropower generation while ensuring domestic water supply for Miaoli County and Taichung City. The result was that the weir diverted as much water as possible from the Daan River to Liyutan, regardless of the water volume (outflow value = B) discharged upstream into the Daan River.

**Figure 4:**
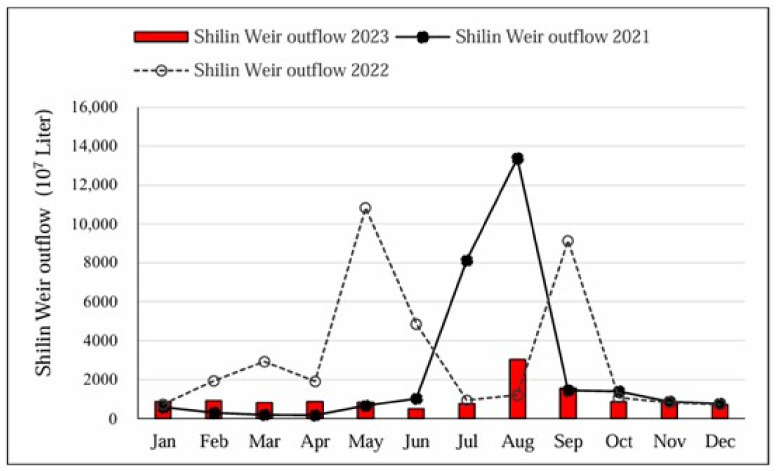
The monthly outflow (×10^4^ m^3^) of Shilin Weir to the Daan River in 2021, 2022, and 2023. Data source: Taiwan Power Company.

Figure 4 indicates that the monthly outflow from the Shilin Weir to Daan River in the upstream area experienced a notable decrease in 2023 compared to the previous two years, causing the riverbed to remain dry throughout the year.

**Figure 5:**
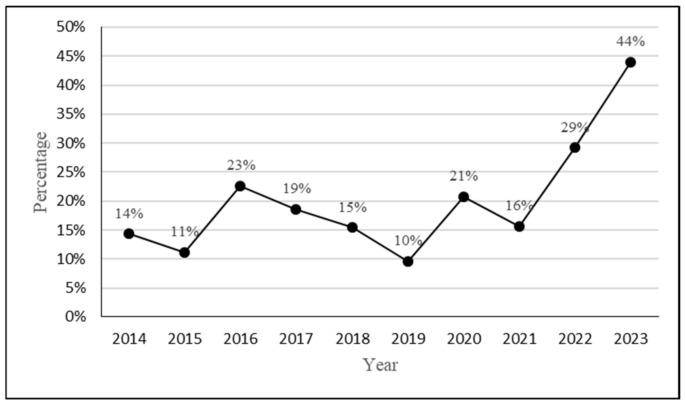
The percentage of the number of roadkill FFBs during the rainy season to the number of the annual total roadkill FFBs from 2014 to 2023. The curve shows that the activity of FFBs in the rainy season elevated unusually in 2022 and 2023, implying that the weather of the rainy season in 2022 and 2023 became drier, forcing FFBs to leave their mountain habitats for forage.

Table 1 shows the annual numbers of roadkill FFBs during the rainy season (from May to September) and the dry season (from October to April) and their percentages relative to the total annual number of roadkill FFBs, from 2014 to 2023 (Supplementary Materials). Surprisingly, the percentage of roadkill FFBs in the rainy season in 2022 and 2023 consistently increased significantly, implying that the outside activities were more active in 2022 and 2023 in the rainy season. Figure 5 shows the curve of the percentage of the number of roadkill FFBs in the rainy season to the annual total roadkill FFBs from year 2014 to 2023, implying that the outside activity of FFBs in the rainy season increased unusually in 2022 and 2023.

**Figure 6:**
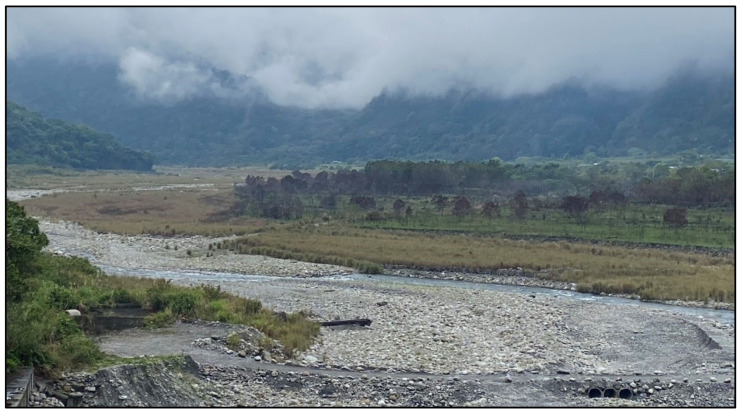
Riverbed conditions at the outflow outlet of the Shilin Weir. The monthly outflow of the Shilin Weir this month is 800 × 10^7^ L (Photo taken by Dr. CH Lai on 19 March 2024).

During site investigations in March 2024, the authors surveyed the dry riverbed in the outflow section of the Shilin Weir on the upstream segment of the Daan River, as shown in Figure 6.

## 4. Discussion

There were several limitations in this study. Under the national animal rabies surveillance programs, the hiring and training of individuals in Miaoli County aimed to trap FFBs for monitoring FFBs rabies. According to trappers, FFBs were trapped mainly in the dry season and were hard to find in the rainy season. This made sampling very difficult during the rainy season. As noted by Mr. Wen-Long Lin, the Chief of the Research Department of Taichung Wildlife Conservation Group, during the rainy season, the surface of the entire mountainous area of Miaoli County was moist, thus FFBs had a lot of prey available, including snails, earthworms, insects, etc. However, during the dry season, the topsoil on the ridge and both sides of the mountainous area dried out, and the prey decreased. This forced FFBs to leave their mountain habitats to forage [12]. Consequently, there were limited samples collected during the rainy season in this study. This phenomenon was also reflected in the proportion of annual roadkill FFBs between the rainy season and the dry season. Table 1 indicates that the annual number of roadkill FFBs in the dry season was always more than in the rainy season in the same year.

Data of the national animal rabies surveillance programs showed that the rabid FFBs invaded into Miaoli County in 2023. The migration of four rabid FFBs into Miaoli County was significant, as the number during the initial phase of a rabies outbreak was typically low [26]. Thus, the presence of four rabid FFBs on the north side of the Daan River proved that FFB rabies had crossed the Daan River. Chiou et al. [2] indicated that from January 2013 to December 2022, a total of 475 FFBs and 196 other wild carnivores were sampled and submitted for rabies surveillance by Miaoli County, all of them were negative for rabies infection which implied that the Daan River indeed blocked the path of the reservoir FFBs and stagnated the spread of the epizootic. Consequently, some factor(s) must have occurred to cause the epizootic to cross the Daan River during year 2022 to 2023.

Climate change in Taiwan is increasingly severe, with the average temperature rising by 1.0~1.4 °C over the past century, surpassing the global average [27]. This significant temperature variation is influenced by various rainfall systems, including the winter monsoon rainy event (WMRE), the frontal rainy event (FRE), and the rainy system north of the South China Sea, illustrating the complexity of precipitation in Taiwan [21]. Kumar et al. [28] demonstrated a positive correlation between rainfall and river runoff. Figure 3 also indicates that higher rainfall in the upstream of the Daan River led to increased runoff and inflow to the Shilin Weir. Conversely, decreased rainfall in the upstream area necessitated a reduction in outflow from the Shilin Weir to the Daan River, maintaining the required water volume in the Liyutan Reservoir for domestic use and hydropower generation. This adjustment sacrificed the upper riverbed of the Daan River, which would cause the upper riverbed of the Daan River to dry up when the water source was insufficient.

Based on the analysis, the level of dryness in the upper riverbed of the Daan River was primarily influenced by the outflow of the Shilin Weir. Figure 3 indicates that the lowest annual outflow of the Shilin Weir occurred in 2023 over an 11-year period from 2013 to 2023. Additionally, Figure 3 also indicates a significant decrease in the annual outflow from the Shilin Weir in 2023. No increase in monthly outflow was recorded during the summer rainy season, indicating consistently low outflow throughout the year. Despite experiencing drought in 2020 [29], Figure 3 reveals high outflows during the rainy season of that year. In contrast, Figure 4 demonstrates consistently low outflow in all months of 2023, which implied that the mountainous area also dried out. This sequentially elevated the outside activity of FFBs for forage.

Rendall et al. [25] indicated that changes in the number of road-killed wildlife were associated with two factors, namely (1) changes in the abundance of wildlife and (2) increased vehicle traffic. In the past two decades, roadkill surveys conducted by citizen scientists have been used to study the distribution of wild animals beyond the limitations of traditionally regular professional observers in traditional survey studies and have thus gradually replaced traditional survey methods. Additionally, since the total length of roads is expected to increase worldwide, the contribution of roadkill survey methods to wildlife studies has experienced sustainable growth [30,31,32,33]. In this study, Table 1 and Figure 5 demonstrate that, from 2014 to 2021, the number of roadkill FFBs accounted for less than 25% of the total number of annual roadkill FFBs during the rainy season. From 2022 to 2023, FFBs had indeed increased activities, even during the typically less active rainy season. Additionally, rainfall data of the year 2023 showed a remarkably low level. These data indicated an extreme lack of rainfall in FFB habitats in the mountains in 2023, forcing FFBs to venture out in search of food and leading to an unusually high number of roadkill FFBs during the rainy season. Zhang et al. [7] found that the activity range for FFBs was approximately 128 hectares, with no gender differences, which provided evidence that the activity range of FFBs was large enough to cross the Daan River. Based on abovementioned information, it was reasonable to conclude that the extremely dry weather in 2023 resulted in FFBs wandering around in search for food, which was the main cause of crossing over the Daan River.

## 5. Conclusions

Recent evidence suggested that FFB rabies had crossed the Daan River, indicating that the river was not a long-term preventive barrier. The hydrological data of this study indicated that low rainfall in the Daan River catchment and river water diversions caused the riverbed to become dry in the upper reaches, allowing FFBs to cross over the Daan river and bring FFB rabies into Miaoli County. Therefore, preventive strategies should focus on amending the contingency plan, maintaining passive surveillance, and assessing the population dynamics and distribution of FFBs to develop effective control policies. These control activities should incorporate high vaccination rates for dogs and cats as well as comprehensive and vigilant surveillance.

## Figures and Tables

**Table 1:** The number of roadkill FFBs during the rainy season and dry season, and their percentages relative to the total annual number of roadkill FFBs, from 2014 to 2023 in Miaoli County.

Year	Rainy Season	Dry Season	Total	Rainy Season (%)	Dry Season (%)
2014	2	12	14	14%	86%
2015	2	16	18	11%	89%
2016	7	24	31	23%	77%
2017	8	35	43	19%	81%
2018	6	33	39	15%	85%
2019	5	47	52	10%	90%
2020	30	115	145	21%	79%
2021	5	27	32	16%	84%
2022	7	17	24	29%	71%
2023	11	14	25	44%	56%

## Data Availability

The data are contained within the article.

## Supplementary Materials

The following supporting information can be downloaded at: https://www.mdpi.com/article/10.3390/ani15030319/s1, Table S1: The data of roadkilled FFBs from 2011 to 2024.
